# Detection and Characterization of Carbapenemase-Producing *Escherichia coli* and *Klebsiella pneumoniae* from Hospital Effluents of Ouagadougou, Burkina Faso

**DOI:** 10.3390/antibiotics12101494

**Published:** 2023-09-29

**Authors:** Alix Bénédicte Kagambèga, René Dembélé, Léa Bientz, Fatima M’Zali, Laure Mayonnove, Alassane Halawen Mohamed, Hiliassa Coulibaly, Nicolas Barro, Véronique Dubois

**Affiliations:** 1 Laboratory of Molecular Biology, Epidemiology and Surveillance of Foodborne Bacteria and Viruses, University Joseph KI-ZERBO of Ouagadougou, Ouagadougou 03 BP 7021, Burkina Faso; mohamed.alassane@outlook.fr (A.H.M.); coulhiliassa@gmail.com (H.C.); nicolas.barro@ujkz.bf (N.B.); 2 Training and Research Unit in Applied Sciences and Technologies, University of Dedougou, Dedougou 03 BP 176, Burkina Faso; 3 UMR 5234, CNRS, Fundamental Microbiology and Pathogenicity, University of Bordeaux, 33000 Bordeaux, France; lea.bientz@u-bordeaux.fr (L.B.); fatima.mzali@u-bordeaux.fr (F.M.); laure.coulange@u-bordeaux.fr (L.M.); veronique.dubois@u-bordeaux.fr (V.D.); 4 Microbiology Laboratory of the General Reference Hospital (GRH), Niamey BP 12674, Niger

**Keywords:** *Escherichia coli*, *Klebsiella pneumoniae*, carbapenemases, hospital effluents, Ouagadougou

## Abstract

Hospital wastewater is a recognized reservoir for resistant Gram-negative bacteria. This study aimed to screen for carbapenemase-producing *Escherichia coli* and *Klebsiella pneumoniae* and their resistance determinants in two hospital effluents of Ouagadougou. Carbapenem-resistant *E. coli* and *K. pneumoniae* were selectively isolated from wastewater collected from two public hospitals in Ouagadougou, Burkina Faso. Bacterial species were identified via MALDI-TOF mass spectrometry. Carbapenemase production was studied phenotypically using antibiotic susceptibility testing via the disk diffusion method. The presence of carbapenemases was further characterized by PCR. A total of 14 *E*. *coli* (13.59%) and 19 *K*. *pneumoniae* (17.92%) carbapenemase-producing isolates were identified with different distributions. They were, respectively, *bla*_NDM_ (71.43%), *bla*_VIM_ (42.86%), *bla*_IMP_ (28.57%), *bla*_KPC_ (14.29%), *bla*_OXA-48_ (14.29%); and *bla*_KPC_ (68.42%), *bla*_NDM_ (68.42%), *bla*_IMP_ (10.53%), *bla*_VIM_ (10.53%), and *bla*_OXA-48_ (5.26%). In addition, eight (57.14%) *E*. *coli* and eleven (57.89%) *K*. *pneumoniae* isolates exhibited more than one carbapenemase, KPC and NDM being the most prevalent combination. Our results highlight the presence of clinically relevant carbapenemase-producing isolates in hospital effluents, suggesting their presence also in hospitals. Their spread into the environment via hospital effluents calls for intensive antimicrobial resistance (AMR) surveillance.

## 1. Introduction

Antimicrobial resistance (AMR) is a growing public health problem worldwide due to the presence of multidrug-resistant (MDR) bacterial pathogens in healthcare settings [1] and their prevalence and persistence in all natural and man-made environments [2]. In 2017, the World Health Organization published a list of pathogens of priority attention, among which are carbapenem-resistant *Enterobacterales* [3]. Carbapenemase-producing *Enterobacterales* (CPE) remain one of the most pressing threats to healthcare [4], as carbapenems are the last resort antimicrobials in clinical settings. Several studies have reported the presence of carbapenemase-producing bacteria in Africa [5], where low- and middle-income countries face a disproportionate burden due to several factors (lack of epidemiological studies and poor diagnostic) that characterize AMR differently than in different countries [5]. Carbapenemases are widespread in many parts of the world [6], and NDM-, KPC-, and OXA-48-like enzymes have become the major mechanism of carbapenem resistance [7], and the most prevalent carbapenemases in the world [8]. *Enterobacterales* such as *Escherichia coli* and *Klebsiella pneumoniae* are common human pathogens and asymptomatic colonizers of the human gastrointestinal tract and environmental niches [9]. *K*. *pneumoniae* and *E. coli* are responsible for various types of infections in humans, including pneumonia, septicemia, and urinary tract infections, in both community and hospital settings [10].

In Burkina Faso, recent surveillance data from the Ministry of Health revealed that in 2018 and 2019, the most frequently isolated bacteria were highly resistant. In *E*. *coli*, resistance levels to penicillin and sulfonamide groups were 90% and over 80%, respectively, in both years. In *Klebsiella* spp., resistance to quinolones was approximately 50%, whereas resistance to third-generation cephalosporins rose from 50% in 2018 to 60% in 2019 [11,12].

Few studies have reported CPEs in the clinical environment of a private hospital in the country, notably carbapenemase-producing *E. coli* (18.87%) [13]. Another study (Markkanen et al.) reported carbapenem resistance genes in the hospital’s wastewater [14]. These findings highlight the need to further screen the environment, and the paucity of data available on the presence and characteristics of CPEs in the environment, particularly in hospital effluents in Burkina Faso, hence the interest of our study. The aim of the study was to identify the presence and characteristics of carbapenemase-producing *E. coli* and *K. pneumoniae* in Ouagadougou hospital effluents. 

## 2. Results

### 2.1. Carbapenemase-Producing E. coli and K. pneumoniae Detection

A total of 209 (77.40%) third-generation cephalosporin-resistant isolates (103 *E*. *coli* and 106 *K. pneumoniae*) were identified from the 270 samples collected during the course of this study by Matrix-Assisted Laser Desorption/Ionization Time-Of-Flight (MALDI-TOF. At the Yalgado Ouedraogo University Hospital (CHU-YO), 28 (27.18%) *E. coli* and 31 (29.24%) *K. pneumoniae* were isolated from raw wastewater. At the Bogodogo University Hospital (CHU-B), 37 (35.92%) *E. coli* and 29 (27.36%) *K. pneumoniae* were isolated from raw wastewater versus 38 (36.90%) *E. coli and* 46 (43.40%) *K. pneumoniae* from treated wastewater (Table 1). Among these isolates, 33 (15.79%) were carbapenem-resistant: 14 (13.59%, 95% CI: 7.63–21.75) *E*. *coli* and 19 (17.92%, 95% CI: 11.15–26.57) *K. pneumoniae* (Table 1).

### 2.2. Antibiotic Resistance Profile 

As expected, a high rate of β-lactam resistance was observed in the isolates. The resistance rate of carbapenemase-producing *E. coli* and *K. pneumoniae* was higher compared to the resistance rate of non-carbapenemase-producing *E. coli* and *K. pneumoniae* (Table 2). This is particularly true for cefoxitin: 85.71% in carbapenemase-producing *E. coli* versus 12.36% in non-carbapenemase-producing *E. coli*; 78.95% in carbapenemase-producing *K. pneumoniae* versus 16.09% in non-carbapenemase-producing *K. pneumoniae*. This resistance was highly plausible, as our selection method targeted third-generation cephalosporine-resistant isolates. However, a high rate of resistance to non-β-lactam antimicrobials was noted: 100% and 85.71% to ciprofloxacin in carbapenemases-producing *K. pneumoniae* and *E. coli*, respectively. For non-carbapenemase producers, ciprofloxacin resistance in *K. pneumoniae* and *E. coli* was 63.2% and 51.7%, respectively. In addition, a very low rate of resistance was observed in amikacin. Interestingly, all carbapenemase-producing *E. coli* and *K. pneumoniae* isolates were resistant to ertapenem, but not to imipenem.

Carbapenemase-producing *E*. *coli* and *K*. *pneumoniae* of the effluents from CHU-YO and CHU-B showed 100% resistance to antibiotics such as ampicillin, piperacillin, cefepime, aztreonam, amoxicillin-clavulanic acid, ceftazidime, and ceftriaxone (Table 3). 

As shown in Table 4, the proportion of MDR isolates was very similar between raw and treated effluents from CHU-B, except that three isolates from the treated wastewater were resistant to 14 antibiotics compared to only one isolate from the raw wastewater. 

The Multiple Antibiotic Resistance (MAR) indices of the effluents from CHU-YO (0.77) and the raw and treated wastewater from CHU-B (0.84 and 0.83, respectively) are above the threshold of 0.2 (Table 5).

### 2.3. Carbapenemase Genes in E. coli and K. pneumoniae 

The carbapenemase genes recovered in *E*. *coli* were 10 *bla*_NDM_ (71.43%), 6 *bla*_VIM_ (42.86%), 4 *bla*_IMP_ (28.57%), 2 *bla*_KPC_ (14.29%), and 2 *bla*_OXA-48_ (14.29%). In *K*. *pneumoniae*, these genes were 13 *bla*_KPC_ (68.42%), 13 *bla*_NDM_ (68.42%), 2 *bla*_IMP_ (10.53%), 2 *bla*_VIM_ (10.53%), and 1 *bla*_OXA-48_ (5.26%). The different genes *detected in E. coli and K. pneumoniae* are presented in supplementary files as follow: *bla*_KPC_*, bla*_VIM_ and *bla*_KPC_ (Supplementary Figure S1), *bla*_NDM_ (Supplementary Figure S2) and *bla*_OXA-48_ (Supplementary Figure S3).

**Figure 1:**
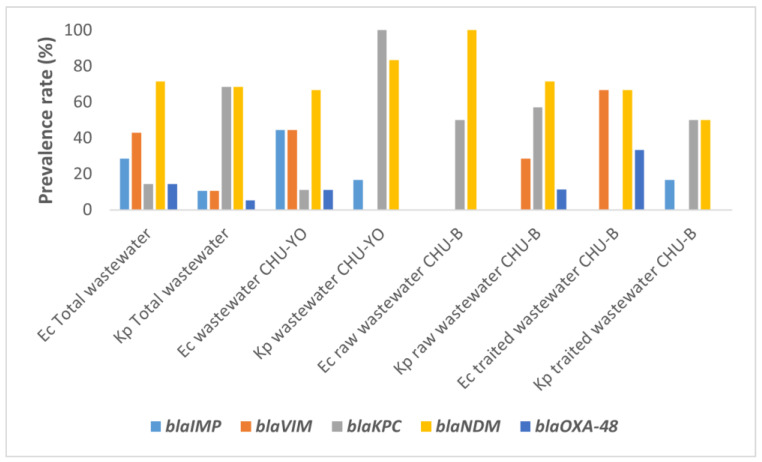
Prevalence of *bla*_IMP_, *bla*_VIM_, *bla*_KPC_, *bla*_NDM_ and *bla*_OXA-48_ in carbapenemase-producing *E. coli* (Ec) and *K. pneumoniae* (Kp).

A high proportion of NDM-producing isolates was observed in each type of effluent. This proportion was 66.67% (95% CI: 29.93–92.51) for raw effluents from CHU-YO, 100% (95% CI: 15.81–100.00) for raw effluents from CHU-B, and 66.67% (95% CI: 9.43–99.16) for treated effluents from CHU-B (Figure 1). IMP- and VIM-producing isolates were detected at 44.44% in CHU-YO effluents, but were not detected in CHU-B effluents untreated for *E. coli* isolates. The proportion of KPC-producing isolates was very high (100%) in CHU-YO wastewater, followed by NDM-producing isolates, whose proportion was higher in CHU-B effluents (100%) (Figure 1).

**Figure 2:**
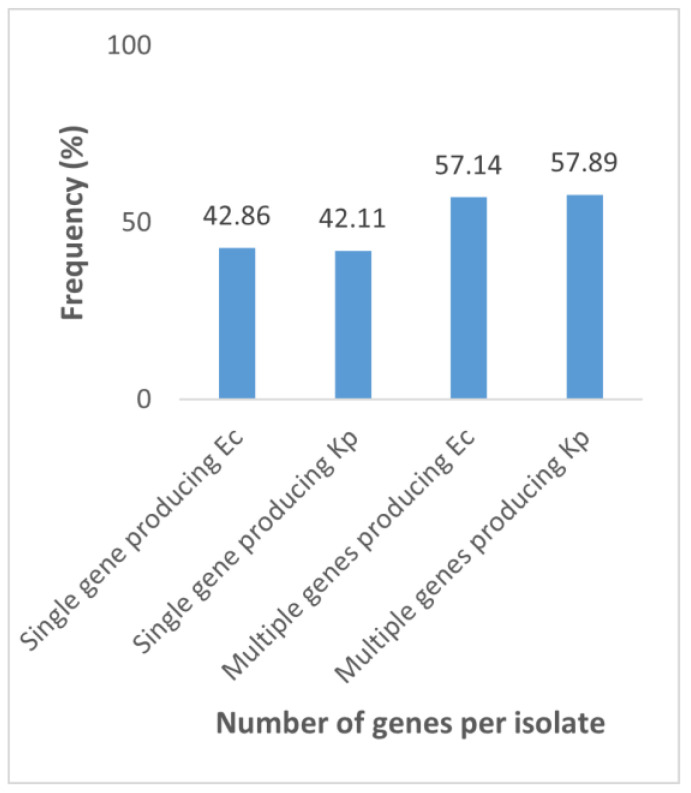
Proportion of the number of genes produced by carbapenemase-producing *E. coli* (Ec) and *K. pneumoniae* (Kp). Single gene producing Ec: *E. coli* producing a single gene; Single gene producing Kp: *K. pneumoniae* producing a single gene; Multiple genes producing Ec: *E. coli* producing multiple genes; Multiple genes producing Kp: *K. pneumoniae* producing multiple genes.

Interestingly, eight (57.14%) *E*. *coli* and eleven (57.89%) *K*. *pneumoniae* isolates (Figure 2) co-produced carbapenemases. Two or more had a high proportion of KPC + NDM (Table 4). However, whether the isolates produced only one or several enzymes at a time, they were resistant to more than nine antibiotics. Moreover, all *bla*_NDM_-producing *E. coli* and *K. pneumoniae* were resistant to aztreonam (Table 4).

## 3. Discussion

We present here a comprehensive study on the occurrence and characteristisation of carbapenemase-producing *E. coli and K. pneumoniae* isolates in wastewater samples over a twelve-month period from two hospitals of Ouagadougou, the capital of Burkina Faso. The study revealed the presence of third-generation cephalosporin-resistant *E. coli* (103/209) and *K. pneumoniae* (106/209) in hospital wastewater samples. The presence of *E. coli* and *K. pneumoniae* could be explained, as these bacterial species are part of the microbiota of the digestive tract of healthy and sick people and are widely distributed in the environment. Hospital effluents are considered a reservoir for multi-drug-resistant pathogens [15,16,17], playing a key role in the spread of antimicrobial-resistant bacteria in the environment [18,19]. Indeed, the present study reports for the first time the presence of carbapenemase-producing *E. coli* and *K. pneumoniae* in hospital wastewater in Ouagadougou with respective prevalence of 13.59% and 17.92%. Similar results were reported in several works including those of Cahill and collaborators who, in 2019, collected 142 isolates of *Enterobacterales* resistant to ertapenem and/or meropenem in hospital effluents [20] and those reported by Zagui and collaborators who, in 2020, isolated strains of *K. pneumoniae* with resistance patterns to broad-spectrum cephalosporins and carbapenems [21]. This proportion of carbapenemase-producing *E. coli* and *K. pneumoniae* in hospital effluents may be explained by a higher frequency of carriage in hospitalized patients than in healthy community carriers [22]. It was shown that the constant load of *E. coli* isolates and the enrichment of *K. pneumoniae* during wastewater treatment may be due to either a shorter generation time of these bacteria or to horizontal transfer of resistance genes [23]. These findings are corroborated by a previous study in Burkina Faso, which reported 18.87% of carbapenemase-producing *E. coli* in clinical settings [13]. The presence of carbapenemase-producing bacteria in the CHU-B raw and treated wastewater highlights the inefficiency of the treatment, which normally should reduce the load of antibiotic-resistant pathogenic bacteria. This situation is worrisome with *Enterobacterales*, especially *E. coli* and *K. pneumoniae*, which are now emerging as difficult-to-treat bacteria [24]. Multidrug resistance of carbapenemase-producing *E. coli* and *K. pneumoniae* was confirmed, and even pan resistance of the latter to almost all antibiotics tested. The resistance patterns of *E. coli* and *K. pneumoniae* were relatively similar between the two hospitals studied. The MAR indices of the raw wastewater from CHU-YO (0.77) and the raw and treated wastewater from CHU-B (0.84 and 0.83, respectively) are higher than the threshold of 0.2, indicating a high risk of environmental contamination and, consequently, public health concerns. The presence of MDR bacteria in hospital effluents could be explained by the release of MDR clinical isolates and the transfer of resistance genes from MDR clinical isolates found in hospital effluents to other environmental bacteria [25]. Moreover, some of the consumed antimicrobials may be excreted unchanged and end up in the environment, contributing to the selection pressure that may lead to the emergence of novel antimicrobial-resistant genes in the environment [26]. Indeed, large amounts of antibiotics are discharged in sewer systems due to incomplete metabolism in humans and sometimes to the disposal of unused antibiotics [27,28]. The presence of disinfectants and antiseptics in sewage can also promote the emergence of antibiotic-resistant bacteria in hospital effluents [29].

The predominance of *bla*_NDM_ recovered in our study in *E. coli* (71.43%) and *K. pneumoniae* (68.42%) is consistent with existing reports [20,30,31,32]. NDM production is a mechanism of carbapenem resistance and is mediated by a ubiquitous plasmid not associated with dominant clonal strains [33]. *E. coli-* and *K. pneumoniae*-producing *bla*_OXA-48_ were also found in hospital wastewater from Algeria [34,35], Tunisia [36], Spain [37], Germany [38], India [39,40], and Brazil [41,42]. This could support the idea that *E. coli-* and *K. pneumoniae*-producing carbapenemases of *bla*_OXA-48_ types (weakly represented in our study) originate from the hospital environment. The presence of carbapenemases producing *E. coli* and *K. pneumoniae* in aqueous environments is a phantom threat [43,44], especially when it is reported from hospital-treated wastewater, as in the case of this study. Recently, inadequate wastewater management by bulk drug manufactures facilities in India led to the contamination of water resources with antimicrobial agents, associated with the selection and dissemination of *bla*_OXA-48_ producers [40]. This means that even after wastewater treatment, significant numbers of carbapenemase-producing bacteria could still survive and be subsequently released into the aquatic environment.

Other genes such as *bla*_VIM_ and *bla*_IMP_ were also recovered from Irish hospitals [20,45]. *bla_VIM_* and *bla*_IMP_ are in higher proportions in *E. coli* (42.86% and 28.57%, respectively) than in *K. pneumoniae* (10.53% and 10.53%, respectively). The high proportion of VIM and, to a lesser extent, IMP in *E. coli* compared with *K. pneumoniae* could be explained by a more rapid diffusion of these genes within *E. coli. bla*_KPC_ was also detected in isolates of hospital wastewater in Tunisia [36], Spain [37], Germany [38], and India [39]. However, the proportion of *bla*_KPC_ was higher in *K. pneumoniae* (68.42%) than in *E. coli* (14.29%). KPC is the most common transmissible class A carbapenemase circulating in *Enterobacterales*, especially in *K. pneumoniae* worldwide, mainly due to the clonal expansion of *K. pneumoniae* strains [46]. These results are somewhat surprising since, according to the first study of hospital wastewater in Burkina Faso using a shotgun metagenomic approach [14], no *bla*_KPC_ was found. 

Analysis of the number of genes expressed by bacterial isolates showed that the majority of our isolates expressed more than one gene, suggesting that isolates harbored multiple plasmid-mediated drug resistant determinants.

Comparison of the antibiotic resistance phenotype with the genotypic profile showed that *E. coli* and *K. pneumoniae* producing only one or several carbapenemases were resistant to more than nine antibiotics, even in wastewater treated before discharge. The emergence and spread of carbapenem resistance in Gram-negative bacilli such as *K. pneumoniae* and *E. coli* via the production of carbapenemases is a global phenomenon. It threatens patient care and could lead to therapeutic failure. This high rate of multi-resistance is of risk as the resistance could spread wider in the environment and attain for instance the food chain. Indeed, a recent study highlighted the presence of resistance genes NDM and VIM among the clinical strains of *E*. *coli* in a hospital in Ouagadougou [13]. Furthermore, these results constitute scientific evidence that could help public authorities, health ministry officials, and the Ouagadougou municipal hygiene department to take appropriate measures to protect and preserve the health of the population. In fact, in the vicinity of the study sites, particularly the CHU-YO, salads and other foodstuffs are produced and consumed by the population.

The potential global spread of critical-priority antimicrobial-resistant *Enterobacterale* is a public health problem. Of course, it was shown that the release of raw and improperly treated wastewater onto water courses has both short- and long-term effects on the environment and human health [47]. Hence, there should be proper enforcement of water and environmental laws to protect the health of inhabitants of both rural and urban communities.

## 4. Materials and Methods

### 4.1. Study Design, Sites and Sampling

This was a cross-sectional study from July 2021 to April 2022 in the CHU-YO and the CHU-B, which are the two most frequented university hospitals in Ouagadougou. Raw wastewater discharged by the CHU-YO flows directly into the city’s sewer system. This is because the hospital has no treatment system. On the other hand, raw wastewater and treated wastewater were collected from CHU-B, which has a functional treatment system. A total of 270 wastewater samples were collected at daily intervals via randomized technique in sterile 250 mL containers: eighty raw wastewater samples from the CHU-YO, ninety-five raw, and ninety-five treated wastewater samples from the CHU-B. The number of samples from CHU-B exceeds that of CHU-YO due to the categorization of its effluents, to lend assiduous credibility to the results that would emanate from the analysis of said effluents. All the samples were transported at <4 °C to the Laboratory of Molecular Biology, Epidemiology and Surveillance of Foodborne Bacteria and Viruses (LaBESTA), within two hours of collection for processing.

### 4.2. Microbial Culturing and Identification

The stock solution was obtained by diluting 10 mL of hospital wastewater in 90 mL of sterile peptone water. A series of decimal dilutions was performed from the stock solution to 1/1000. The following agar plates were inoculated from 1/1000 dilution. First, Chromocult^®^ Coliform Agar (CCA) (Laboratorios Conda S.A), and then Plate Count Agar (PCA) + Tri-phenyl Tetrazolium Chloride (TTC) agar supplemented with ceftriaxone to maximize isolation of extended-spectrum beta-lactamase-producing strains (after CCA exhaustion). Plates were incubated at 37 °C for 24 h. Presumptive colonies were then transferred to Eosin Methylene Blue (EMB) agar (Laboratorios Conda S.A) at 37 °C for 24 h for purification and conservation. Muller Hinton (MH) agar (Laboratorios Conda S.A r) was used to subculture the isolates at 37 ± 0.5 °C for 24 h and confirm the identification via MALDI-TOF mass spectrometry using the Microflex MALDI-TOF MS^®^ (Bruker Daltonics, Bremen, Germany) equipment. As the identification of *E*. *coli* with MALDI-TOF was not accurate (confusion between *E*. *coli* and *Shigella*), all *E. coli* identified by MALDI-TOF were confirmed on Bromocresol Purple (BCP) Agar plate and subsequent API 20E gallery (bioMerieux, Marcy-l’Etoile France) for lactose negative isolates.

### 4.3. Antibiotic Susceptibility Testing

Antimicrobial susceptibility testing to 16 antibiotics was determined via the agar disk diffusion method on MH agar plates according to the Antimicrobial Susceptibility Testing Committee of the French Microbiology Society/European Committee on Antimicrobial Susceptibility Testing (CA-SFM/EUCAST), 2021 guidelines [48]. The following antibiotics (MAST Amiens France) were used: Ampicillin (AMP) 10 μg, Piperacillin (PIP) 30 μg, Piperacillin-Tazobatam (PTZ) 36 μg, Amoxicillin-Clavulanic acid (AUG) 30 μg, Aztreonam (ATM) 30 μg, Imipenem (IMP) 10 μg, Ertapenem (ETP) 10 μg, Ceftriaxone (CRO) 30 μg, Cefoxitin (FOX) 30 μg, Cefepime (FEP) 30 μg, Ceftazidime (CAZ) 30 μg, Gentamicin (GM) 10 μg, Amikacin (AK) 30 μg, Nalidixic acid (NA) 30 μg, Ciprofloxacin (CIP) 5 μg, and Trimethoprim-Sulfamethoxazole (SXT) 25 μg). Antibiotic susceptibility was interpreted according to the CA-SFM/EUCAST, 2021 guidelines recommendations [48]. For isolates with reduced susceptibility to carbapenems (ertapenem), DNA was extracted for carbapenemase genes. For quality control, we used *E. coli* ATCC 25922 and *K. pneumoniae* ATCC 700603, which produces an ESBL (SHV-18).

The Multiple Antibiotic Resistance (MAR) Index per sampling site was calculated using the Krumperman method [49] to compare the contribution of each human activity to the risk to the environment and public health. Fifteen of the antibiotics tested were included in the index calculation, with CRO excluded because the medium used to select isolates was CRO-supplemented. 

Sites with an index of 0.2 or less were considered to present a low risk of contamination because antibiotics are rarely or never used, whereas sites with an index greater than 0.2 were considered to present a high risk of contamination because of constant and significant exposure to antibiotics.

### 4.4. Detection of Carbapenemase Genes

DNA was extracted from *E. coli* or *K. pneumoniae* isolates using organic extraction (phenol–chloroform method) as previously described [50]. The quantity, purity, and integrity of the extracted DNA were verified via 1% agarose gel electrophoresis and nanodrop measurement. 

Multiplex PCR was performed for the detection of carbapenemase genes (*bla*_IMP_, *bla*_VIM_, *bla*_KPC_, *bla*_NDM,_ and *bla*_OXA-48_) using primers previously described [51,52]. Five μL of sample DNA [C < 250 ng, https://www.promega.com.au/-/media/files/resources/protocols/product-information-sheets/g/gotaq-hot-start-green-master-mix-protocol.pdf, accessed on 28 August 2023] was subjected to each multiplex PCR in a 25 μL reaction mixture containing PCR buffer (5X, primers (10 µM), and PCR water using the GoTaq^®^ Master mixes (Promega, Charbonnières-les-Bains, France). Amplification was performed as follows: initial denaturation at 95 °C for 3 min; 34 cycles of 95 °C for 40 s, 55 °C for 40 s, and 72 °C for 1 min; and a final elongation step at 72 °C for 5 min. For amplification of *bla*_OXA-48_ genes, the annealing temperature was optimal at 60 °C. Amplicons were visualized on a 1% agarose gel containing ethidium bromide. For quality control, we used clinical strains obtained from the Pelegrin Hospital of Bordeaux, for which the genes were sequenced [50].

## 5. Conclusions

This study constitutes the first report of enterobacterial carbapenemase producers in hospital wastewater in Burkina Faso. One limitation of our study is that we did not have the opportunity to confirm the identity of carbapenemase genes via the DNA sequencing technique. In the future, it would be interesting to extract the potential plasmids carrying these genes and pass them into reference strains of *E*. *coli* by transformation (TOP10 for example) or by conjugation (*E*. *coli* K12). The high level of resistance in these strains, particularly to carbapenems, shows that at least one of the two genes is expressed. Indeed, it is well known that the rapid and global dissemination of critical-priority antimicrobial-resistant *Enterobacterales* is a public health problem that demands mitigation strategies and strengthening via epidemiological surveillance investigations. However, the results of the study highlight the danger to public health posed by hospital effluents to the population of Ouagadougou and show that measures need to be taken to protect public health. A significant occurrence of carbapenemase *E*. *coli* and *K*. *pneumoniae* was detected in this study, suggesting that hospital effluents may act as a potential reservoir of MDR bacterial pathogens and play a key role in their dissemination in the environment that can negatively impact human health. Indeed, our results show very high levels of carbapenemase genes including *bla*_NDM_, *bla*_VIM_, *bla*_IMP_, *bla*_KPC_, and *bla*_OXA-48_ in both *E*. *coli* and *K*. *pneumoniae* strains. This spread of carbapenemase-producing *Enterobacterales* in hospital wastewater warrants the need for intensive surveillance of AMR and the implementation of an efficient infection control program in Burkina Faso for the management of such infections.

## Figures and Tables

**Table 1:** The rate of detection of carbapenemase producing *E*. *coli* and *K*. *pneumoniae*.

Sampling Sites	Wastewater Type	*E*. *coli n* (%)	*K*. *pneumoniae n* (%)	Total Number *n* (%)
CHU-YO	Raw	9/28 (32.14)	6/31 (19.35)	15/59 (25.42)
CHU-B	Raw	2/37 (5.41)	7/29 (24.14)	9/66 (13.64)
	Treated	3/38 (7.89)	6/46 (13.04)	9/84 (10.71)
Total number *n* (%)		14/103 (13.59)	19/106 (17.92)	33/209 (15.79)

**Table 2:** Resistance rate of non-carbapenemases-producing and carbapenemase-producing isolates.

	AMC	AMP	PIP	PTZ	FOX	CRO	CAZ	FEP	ATM	ETP	IMP	AN	CIP	SXT	GM	AK
NCPEc	39.33	100	98.88	5.62	12.36	83.15	79.78	75.53	79.78	3.4	0	58.43	51.69	69.66	28.09	2.25
CPEc	100	100	100	78.57	92.86	100	100	100	100	100	0	71.43	85.71	85.71	28.57	7.14
NCPKp	31.03	100	95.51	4.6	16.09	74.71	73.56	66.67	67.82	14	0	21.84	63.22	79.31	44.83	1.15
CPKp	100	100	100	73.68	78.95	100	100	100	100	100	0	31.58	100	94.74	68.42	5.26

NCPEc: Non-Carbapenemase-producing *E. coli*; CPEc: Carbapenemase-producing *E. coli*; NCPKp: Non-Carbapenemase-producing *K. pneumoniae* and CPKp: Carbapenemase-producing *K. pneumoniae.* AMP: Ampicillin, PIP: Piperacillin, PTZ: Piperacillin-Tazobactam, AUG: Amoxicillin-Clavulanic acid, ATM: Aztreonam, IMP: Imipenem, ETP: Ertapenem, CRO: Ceftriaxone, FOX: Cefoxitin, FEP: Cefepime, CAZ: Ceftazidime, CN: Gentamicin, AK: Amikacin, NA: Nalidixic acid, CIP: Ciprofloxacin, SXT: Trimethoprim-Sulfamethoxazole.

**Table 3:** Carbapenemase-producing isolates according to the origin and nature of the effluents.

Sampling Sites	Wastewater Type	Antibiotics	AMP	PIP	SXT	FOX	GM	PTZ	FEP	ETP	AK	ATM	AMC	CAZ	AN	CIP	CRO	IMP
		Isolates																
CHU-YO	Raw	CPEc	100	100	88.88	100	44.44	77.77	100	100	0	100	100	100	77.77	88.88	100	0
		CPKp	100	100	85.71	83.33	50	50	100	100	0	100	100	100	0	100	100	0
CHU-B	Raw	CPEc	100	100	50	50	0	50	100	100	50	100	100	100	50	100	100	0
		CPKp	100	100	100	100	85.71	100	100	85.71	0	100	100	100	28.57	100	100	0
	Treated	CPEc	100	100	100	100	0	1006	100	100	0	100	100	100	66.67	66.68	100	0
		CPKp	100	100	83.33	50	83.33	66.67	100	100	0	100	100	100	66.67	100	100	0

CPEc: Carbapenemase-producing *E. coli* and CPKp: Carbapenemase-producing *K. pneumoniae.*

**Table 4:** Antibiotic resistance phenotype and carbapenemase genes harbored by *E. coli* and *K. pneumoniae*.

Sampling Sites	MALDI Identification	Antibiotic Resistance Phenotype	Resistance Genes
	*K. pneumoniae*	AMP-PIP-SXT-FOX-PTZ-FEP-ETP-ATM-AMC-CAZ-CIP-CRO	KPC-NDM
	*K. pneumoniae*	AMP-PIP-SXT-FOX-GM-FEP-ETP-ATM-AMC-CAZ-CIP-CRO	IMP-KPC-NDM
	*K. pneumoniae*	AMP-PIP-SXT-FOX-GM-FEP-ETP-ATM-AMC-CAZ-CIP-CRO	KPC-NDM
	*K. pneumoniae*	AMP-PIP-SXT-GM-FEP-ETP-ATM-AMC-CAZ-CIP-CRO	KPC-
	*K. pneumoniae*	AMP-PIP-SXT-FOX-PTZ-FEP-ETP-ATM-AMC-CAZ-CIP-CRO	KPC-NDM
	*K. pneumoniae*	AMP-PIP-SXT-FOX-PTZ-FEP-ETP-ATM-AMC-CAZ-CIP-CRO	KPC-NDM
CHU-YO	*E. coli*	AMP-PIP-SXT-FOX-PTZ-FEP-ETP-ATM-AMC-CAZ-AN-CIP-CRO	VIM-NDM
	*E. coli*	AMP-PIP-SXT-FOX-PTZ-FEP-ETP-ATM-AMC-CAZ-AN-CIP-CRO	VIM-NDM
	*E. coli*	AMP-PIP-SXT-FOX-GM-PTZ-FEP-ETP-ATM-AMC-CAZ-AN-CIP-CRO	IMP-NDM
	*E. coli*	AMP-PIP-SXT-FOX-GM-PTZ-FEP-ETP-ATM-AMC-CAZ-CIP-CRO	KPC-NDM
	*E. coli*	AMP-PIP-SXT-FOX-GM-PTZ-FEP-ETP-ATM-AMC-CAZ-AN-CIP-CRO	IMP-OXA48
	*E. coli*	AMP-PIP-FOX-FEP-ETP-ATM-AMC-CAZ-CRO	IMP
	*E. coli*	AMP-PIP-SXT-FOX-GM-FEP-ETP-ATM-AMC-CAZ-AN-CIP-CRO	IMP
	*E. coli*	AMP-PIP-SXT-FOX-PTZ-FEP-ETP-ATM-AMC-CAZ-AN-CIP-CRO	VIM-NDM
	*E. coli*	AMP-PIP-SXT-FOX-PTZ-FEP-ETP-ATM-AMC-CAZ-AN-CIP-CRO	VIM-NDM
	*K. pneumoniae*	AMP-PIP-SXT-FOX-GM-PTZ-FEP-ETP-ATM-AMC-CAZ-CIP-CRO	KPC-NDM
	*K. pneumoniae*	AMP-PIP-SXT-FOX-GM-PTZ-FEP-ETP-ATM-AMC-CAZ-AN-CIP-CRO	KPC
	*K. pneumoniae*	AMP-PIP-SXT-FOX-GM-PTZ-FEP-ETP-ATM-AMC-CAZ-CIP-CRO	VIM-NDM
	*K. pneumoniae*	AMP-PIP-SXT-FOX-GM-PTZ-FEP-ETP-ATM-AMC-CAZ-CIP-CRO	KPC-NDM
	*K. pneumoniae*	AMP-PIP-SXT-FOX-GM-PTZ-FEP-ETP-ATM-AMC-CAZ-CIP-CRO	KPC-NDM
	*K. pneumoniae*	AMP-PIP-SXT-FOX-GM-PTZ-FEP-ETP-ATM-AMC-CAZ-CIP-CRO	NDM
	*K. pneumoniae*	AMP-PIP-SXT-FOX-PTZ-FEP-ETP-ATM-AMC-CAZ-AN-CIP-CRO	VIM-OXA48
	*K. pneumoniae*	AMP-PIP-FEP-ETP-ATM-AMC-CAZ-AN-CIP-CRO	NDM
CHU-B	*K. pneumoniae*	AMP-PIP-SXT-FOX-GM-PTZ-FEP-ETP-ATM-AMC-CAZ-AN-CIP-CRO	NDM
	*K. pneumoniae*	AMP-PIP-SXT-GM-FEP-ETP-ATM-AMC-CAZ-CIP-CRO	KPC
	*K. pneumoniae*	AMP-PIP-SXT-GM-PTZ-FEP-ETP-AK-ATM-AMC-CAZ-AN-CIP-CRO	IMP
	*K. pneumoniae*	AMP-PIP-SXT-FOX-GM-PTZ-FEP-ETP-ATM-AMC-CAZ-CIP-CRO	KPC-NDM
	*K. pneumoniae*	AMP-PIP-SXT-FOX-GM-PTZ-FEP-ETP-ATM-AMC-CAZ-AN-CIP-CRO	KPC
	*E. coli*	AMP-PIP-SXT-FOX-PTZ-FEP-ETP-ATM-AMC-CAZ-CIP-CRO	NDM
	*E. coli*	AMP-PIP-FEP-ETP-AK-ATM-AMC-CAZ-AN-CIP-CRO	KPC-NDM
	*E. coli*	AMP-PIP-SXT-FOX-PTZ-FEP-ETP-ATM-AMC-CAZ-AN-CIP-CRO	VIM-NDM
	*E. coli*	AMP-PIP-SXT-FOX-PTZ-FEP-ETP-ATM-AMC-CAZ-AN-CIP-CRO	VIM-OXA48
	*E. coli*	AMP-PIP-SXT-FOX-PTZ-FEP-ETP-ATM-AMC-CAZ-CRO	NDM

**Table 5:** Multiple Antibiotic Resistance (MAR) index of wastewater samples.

Sampling Sites	Wastewater Type	Number of Isolates	N. Antibiotics	MAR Index
CHU-YO	Raw	15	185	0.77
CHU-B	Raw	9	114	0.84
	Treated	9	112	0.83

N. Antibiotics: sum of the number of antibiotic resistances per isolate.

## Data Availability

No new data were created.

## Supplementary Materials

The following supporting information can be downloaded at: https://www.mdpi.com/article/10.3390/antibiotics12101494/s1, Figure S1: *bla*_KPC_*, bla*_VIM_ and *bla*_KPC_ genes detected in *E. coli* and *K. pneumoniae*; Figure S2: *bla*_NDM_ gene detected in *E. coli* and *K. pneumoniae;* Figure S3: *bla*_OXA-48_ gene detected in *E. coli* and *K. pneumoniae.*
