# Supplementary material for: Detection and Characterization of Carbapenemase-Producing Escherichia coli and Klebsiella pneumoniae from Hospital Effluents of Ouagadougou, Burkina Faso
Source: Antibiotics (Basel). 2023 Sep 29;12(10):1494. doi: 10.3390/antibiotics12101494 (PMC10603891; doi:10.3390/antibiotics12101494)
Supplement: Supplementary file 1 [file antibiotics-12-01494-s001.zip › antibiotics-2559406-supplementary.pdf]

Supplementary Materials

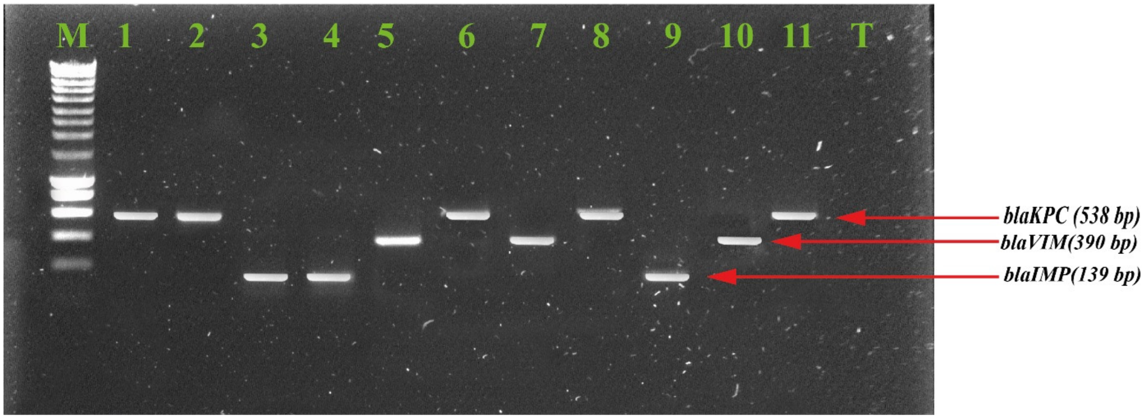

Figure S1: *blaKPC, blaVIM and blaKPC genes detected in E. coli and K. pneumoniae.*

Figure legend : Lane M = hyperlader 100 bp; Lane 3 and 4 = positive samples for *blaIM*P gene (139 pb), Lane 5 and 7 = positive samples for *blaVIM* gene (390 pb), Lane 1 2 6 and 8 = positive samples for *blaKPC* gene (538 pb), Lane 9 = *blaIMP* positive control (139 pb), Lane 10 = *blaVIM* positive control (390 pb), Lane 11 = *blaKPC* positive control (538 pb), T: *blaKPC* negative control (538 pb),

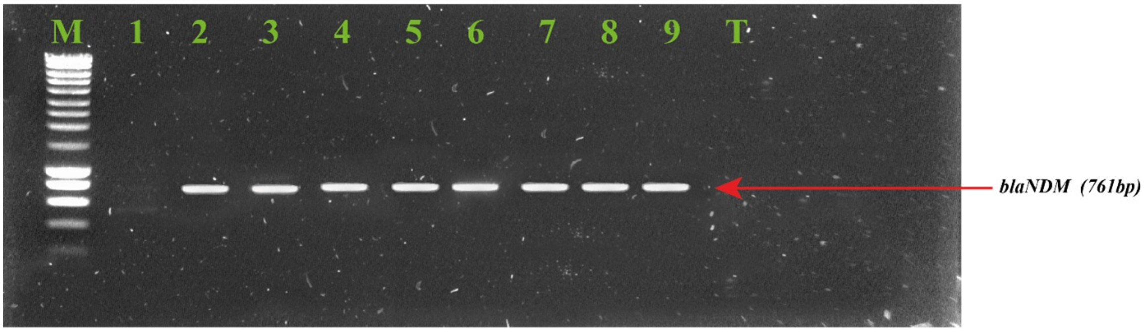

Figure S2: *blaNDM gene detected in E. coli and K. pneumoniae.*

Figure legend: Lane M = hyperlader (100 bp); Lane 1 = negative sample for *blaNDM* gene (761 pb), Lane 2 ;3 ;4 ;5 ;6 ;7 ; and 8 = positive samples for *blaNDM* gene (761 pb), Lane 9 = *blaNDM* positive control (761 pb), T: *blaNDM* negative control (761 pb).

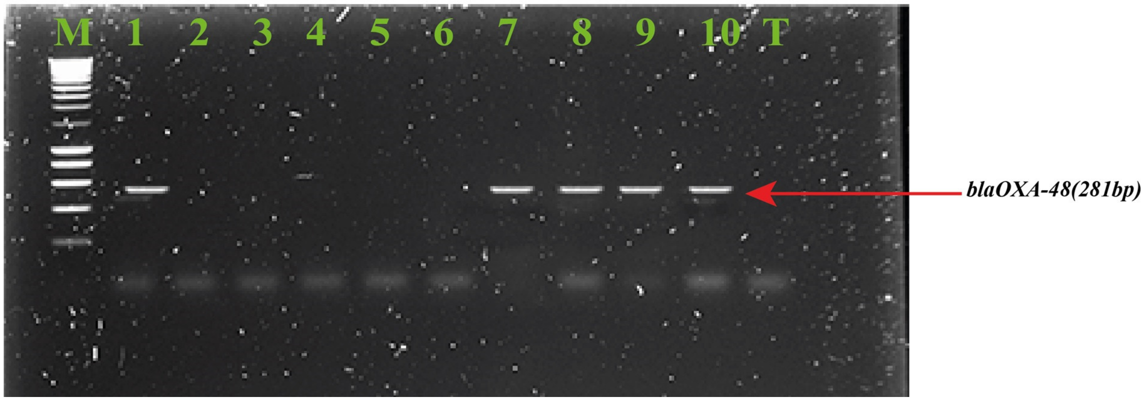

Figure S3: *blaOXA-48 gene detected in E. coli and K. pneumoniae. .*

Figure legend: Lane M = hyperlader (100 bp); Lane 2 ;3 ;4 ;5 and 6 = negative sample for *blaOXA-48* gene (281 pb), Lane 1 ;7 ;8 and 9 = positive samples for *blaOXA-48* gene (281 pb), Lane 10 = *blaOXA-48* positive control (281 pb), T: *blaOXA-48* negaive control (281 pb).
